# DFT and Molecular Docking Study of HA-Conjugated SWCNTs for CD44-Targeted Delivery of Platinum-Based Chemotherapeutics

**DOI:** 10.3390/ph18060805

**Published:** 2025-05-27

**Authors:** Muhammad Uzair Khan, Ishrat Jabeen, Abdulhamid Althagafi, Muhammad Umar Farooq, Moussab Harb, Bassim Arkook

**Affiliations:** 1 School of Interdisciplinary Engineering and Sciences, National University of Sciences and Technology, Islamabad 44000, Pakistan; muzair.mscse24ssines@student.nust.edu.pk; 2 Pharmacy Practice Department, Faculty of Pharmacy, King Abdulaziz University, Jeddah 21589, Saudi Arabia; aalthagfi@kau.edu.sa; 3 State Key Laboratory of Synergistic Chem-Bio Synthesis, School of Chemistry and Chemical Engineering, Shanghai Jiao Tong University, Shanghai 200240, China; umar@sjtu.edu.cn; 4 Department of Physics, Faculty of Science, King Abdulaziz University, Jeddah 21589, Saudi Arabia; mharb@kau.edu.sa

**Keywords:** hyaluronic acid, carbon nanotubes, platinum-based drugs, density functional theory, molecular docking, CD44 receptor

## Abstract

**Background:** Hyaluronicacid (HA)-conjugated nanocarriers leverage CD44 receptor overexpression on tumor cells for targeted delivery of platinum chemotherapeutics. **Methods:** This study compares non-functionalized (DDS1) versus HA-conjugated single-walled carbon nanotubes (DDS2) for encapsulation stability and CD44 binding of Cisplatin, Carboplatin, and Lobaplatin. Density Functional Theory calculations employed PBE-GGA with Tkatchenko–Scheffler dispersion and ZORA relativistic treatment, using a finite (8,8) armchair SWCNT (24.6 Å, H-capped) for DDS1 and an EDC/NHS-coupled HA oligomer for DDS2. We computed binding energies, HOMO–LUMO gaps, Molecular Electrostatic Potentials, and energy decompositions. Molecular docking to CD44 (PDB ID: 4PZ3) used Molegro Virtual Docker, validated by re-docking the native HA fragment (RMSD 1.79 Å). **Results:** DFT binding energies (eV) for DDS2 versus DDS1 were −7.92/−7.48 (Cisplatin), −8.93/−8.30 (Carboplatin), and −9.72/−9.25 (Lobaplatin), indicating enhanced stabilization by HA functionalization. Energy decomposition showed increases of ∼0.4 eV (vdW) and ∼0.2 eV (electrostatic) in DDS2. MEP maps confirmed additional negative-potential regions on DDS2, complementing drug-positive sites. Molecular docking yielded MolDock scores of −171.26 for DDS2 versus −106.68 for DDS1, reflecting stronger CD44 affinity. Docking scores indicate that HA conjugation notably strengthens the predicted affinity of CNT carriers toward the CD44 receptor (ΔScore ≈ −65 kcal mol^−1^). **Conclusions:** These results motivate experimental follow-up to confirm whether DDS2 can translate the in silico affinity gains into improved targeted delivery of platinum chemotherapeutics.

## 1. Introduction

Hyaluronic acid (HA) is a naturally occurring biopolymer widely recognized for its biocompatibility, biodegradability, and ability to selectively bind to CD44 receptors, which are overexpressed in various tumor cells [1,2,3,4]. The structural diversity of CD44’s hyaluronan-binding domains, as documented across multiple crystallographic entries (see Table S1 in Supplementary Material), highlights the complexity and specificity of HA-mediated targeting.

Previous studies have shown that the molecular weight (MW) of HA critically influences CD44-mediated uptake. Polymers with an average MW of approximately 200 kDa are reported to achieve optimal receptor clustering and tumor accumulation, while significantly lower or higher molecular weights can diminish targeting efficacy due to insufficient multi-valency or steric hindrance [1,3]. The critical importance of HA MW in determining nanoparticle-cell interactions and subsequent biological outcomes is further underscored by research investigating various HA MWs. For instance, Chiesa et al. demonstrated that HA MW (ranging from 280 kDa to 820 kDa) significantly impacts the endocytic mechanisms—such as clathrin-mediated versus caveolae-mediated pathways—utilized by HA-based nanoparticles for cellular entry, as well as influencing cell proliferation, even when nanoparticle size was kept consistent [5]. Their work highlights that the choice of HA MW is a key determinant of how nanocarriers interact with CD44-expressing cells. Furthermore, other studies, such as that by Della Sala et al., have specifically investigated 200 kDa HA in the formulation of CD44-targeted nanoparticles, noting MW-dependent differences in uptake kinetics [6]. Based on these collective findings, which emphasize a balance between effective receptor engagement and predictable biological interactions, we employ HA with an average MW of 200 kDa in this study.

Among emerging nano drug delivery systems (DDSs), HA-conjugated single-walled carbon nanotubes (SWCNTs) have demonstrated significant potential. SWCNTs offer several advantages over multi-walled carbon nanotubes (MWCNTs), including a higher surface-to-volume ratio, well-defined monolayer structures, and a narrow internal diameter (∼10.85 Å) that matches small-molecule drug dimensions [7,8,9,10]. These properties enable efficient encapsulation, improved functionalization, and favorable biological interactions. Furthermore, HA conjugation enhances the hydrophilicity and dispersion stability of SWCNTs, reducing aggregation and mitigating cytotoxicity risks associated with CNTs [11,12].

Despite extensive research on either hyaluronic acid (HA)-functionalized nanoparticles [1,3,13] or pristine/surface-modified carbon nanotubes (CNTs) for anticancer therapy [7,9], a unified platform that simultaneously (i) preserves the high drug-loading capacity of single-walled CNTs (SWCNTs), (ii) leverages active CD44 targeting through HA, and (iii) quantitatively benchmarks its performance at the electronic structure level remains absent. Previous HA–nanocarriers (e.g., liposomes, micelles, dendrimers) suffer from limited mechanical robustness and low in situ loading of small, rigid molecules such as platinum drugs. Our HA–SWCNT construct (DDS2) provides an inner graphitic cavity for π–π/van der Waals confinement and an outer HA corona for CD44 recognition, achieving >10-fold higher predicted binding affinity to CD44 (64 kcal/mol improvement vs. state-of-the-art HA-liposomes [2]). To our knowledge, this is the first study coupling ZORA-relativistic DFT (capturing Pt–ligand covalency) with ensemble-corrected MolDock/MM-GBSA docking to dissect how HA conjugation modulates both *drug encapsulation thermodynamics* (up to 0.6 eV stronger) and *receptor binding* (ΔG_bind_≈−9.3 kcal/mol). Earlier CNT reports relied mostly on force-field MD without electronic validation, leaving the Pt–CNT interaction strength speculative. We design a 10-disaccharide HA oligomer that (a) retains essential CD44 epitope motifs yet (b) avoids steric blocking of the SWCNT lumen—an issue noted in high-MW HA coatings [4]. Control calculations with longer chains confirm minimal scoring benefit but increased steric hindrance. By benchmarking Cisplatin, Carboplatin, and Lobaplatin in the same nanocarrier, we show how carrier electronics and sterics govern a universal loading trend (LBP > CBP > CP). No prior HA or CNT study has provided such comparative insight across the three clinically dominant Pt drugs. Mulliken/Hirshfeld analyses reveal negligible charge transfer, suggesting that DDS2 maintains the pro-drug form until triggered release, potentially mitigating the premature Pt–protein adduct formation reported for bare CNT vectors [10]. Collectively, these advances position HA-conjugated SWCNTs as a next-generation, electronically validated vehicle that bridges the loading efficiency of CNTs with the tumor selectivity of HA, surpassing the limitations of existing single-function systems.

The platinum panel adopted herein—Cisplatin (first-generation), Carboplatin (second-generation), and Lobaplatin (third-generation)—was chosen for three complementary reasons: (1) Global clinical relevance. Cisplatin and Carboplatin remain first-line chemotherapeutics for a broad spectrum of solid tumors, collectively accounting for ∼70% of platinum prescriptions worldwide [14]. Lobaplatin, while approved mainly in Asia, represents the most advanced FDA-orphan-designated analog with proven efficacy against cisplatin-resistant malignancies [15]. Focusing on these agents, therefore, maximizes translational impact. (2) Incremental structural diversity within a constant coordination sphere. All three drugs share the classic Pt(II) (diamine) core but differ systematically in their leaving-group chemistry—chlorido (Cisplatin), bidentate dicarboxylate (Carboplatin), and cyclobutanedicarboxylate + lactic acid ligand set (Lobaplatin). This progression allows us to interrogate how increasing steric bulk and hydrophilicity modulate SWCNT encapsulation and HA-mediated targeting while keeping the electronic environment of Pt(II) comparable—an essential requirement for meaningful DFT benchmarking. (3) Pragmatic exclusion of other analogs. *Oxaliplatin* contains a bulky 1,2-diaminocyclohexane ligand whose diameter (≈6.6 Å) approaches the inner radius of the (8,8) SWCNT, producing unstable docking poses and artificially high strain energies in pilot calculations. *Satraplatin* and *Picoplatin* are Pt(IV) or sterically hindered pro-drugs that require metabolic reduction; modeling their bioactivation lies beyond the mechanistic scope of the present work. Regionally restricted analogs such as *Nedaplatin* or *Heptaplatin* show limited global use and lack comprehensive CD44-expression response data, precluding rigorous in silico validation. Consequently, the selected triad balances clinical prevalence, mechanistic representativeness, and computational tractability, forming a coherent testbed for evaluating the added value of HA-conjugated SWCNT delivery.

Although HA’s carboxyl groups have a low p$K_{a}$ (∼3–4) [16,17,18], making them largely deprotonated even at tumor-like pH levels (6.5–6.8) [19], significant drug release from HA-based systems is more likely to occur within acidic intracellular compartments (e.g., endosomes and lysosomes, pH 5.0–5.5) [16,20,21] or through enzymatic degradation by hyaluronidase, which is often overexpressed in tumor tissues [21,22], rather than through pH-triggered mechanisms alone at the slightly acidic tumor extracellular pH.

Platinum-based chemotherapeutics such as Cisplatin, Carboplatin, and Lobaplatin are among the most widely used anticancer agents but suffer from dose-limiting toxicities and the emergence of drug resistance [13,14,15,23]. Encapsulation within HA-conjugated SWCNTs offers a strategy to enhance targeted delivery, minimize off-target effects, and improve therapeutic efficacy.

Computational modeling, combining Density Functional Theory (DFT) and molecular docking, provides valuable atomistic insights into the design and optimization of DDSs. DFT enables accurate predictions of drug encapsulation and stability by modeling electronic structures [24], while molecular docking simulates interactions between DDSs and biological targets such as CD44 receptors, offering estimates of binding affinities and targeting efficiency [25].

Cutting-edge studies published during the past two years substantiate–and contextualize–the rationale for our HA-SWCNT platform: HA nanocarriers: Novel HA-derived pro-drug nanomedicines and lipid nanocarriers have achieved CD44-guided tumor accumulation but still suffer from limited payload density for rigid metallodrugs [12,26]. CNT delivery of platinum agents: 2024 reviews report significant strides in CNT-mediated Pt delivery yet emphasize the need for active-targeting coronas to overcome off-target toxicity [27,28]. Dual-target or combination constructs: A 2024 CD44-targeted HA/Cisplatin nanogel displayed potent synergy in NSCLC, highlighting the clinical momentum for CD44-driven platinum therapy [29]. HA conjugation to emerging carbon nanomaterials: Recent work on HA-functionalized nanodiamonds reinforces the generality of HA corona engineering for carbon-based vectors [30]. These reports collectively underscore a clear knowledge gap: no prior study has quantitatively dissected, at the electronic structure level, how HA conjugation modulates both platinum drug encapsulation and CD44 affinity of CNT carriers–precisely the focus of our present work.

In this study, we employ DFT and molecular docking approaches to investigate the encapsulation properties, stability, and receptor-targeting interactions of platinum-based anticancer drugs within two SWCNT-based DDSs: non-functionalized SWCNTs (DDS1) and HA-conjugated SWCNTs (DDS2). Specifically, we aim to achieve the following:

- Evaluate the stability and encapsulation efficiency of drug-loaded DDS1 and DDS2 via DFT calculations,
- Analyze the binding affinity and interaction profiles of DDSs with the CD44 receptor through molecular docking simulations,
- Assess the influence of HA conjugation on the targeting specificity and biocompatibility of SWCNT-based DDSs.

Our findings offer theoretical insights that can guide the development of HA-conjugated SWCNT drug delivery platforms for platinum-based chemotherapy, aiming to enhance therapeutic outcomes while minimizing systemic side effects.

## 2. Results

### 2.1. Molecular Structures of Investigated Compounds

The molecular structures of the primary compounds investigated in this study include hyaluronic acid (HA), the carrier system components, and three platinum-based anticancer drugs.

**Figure 1:**
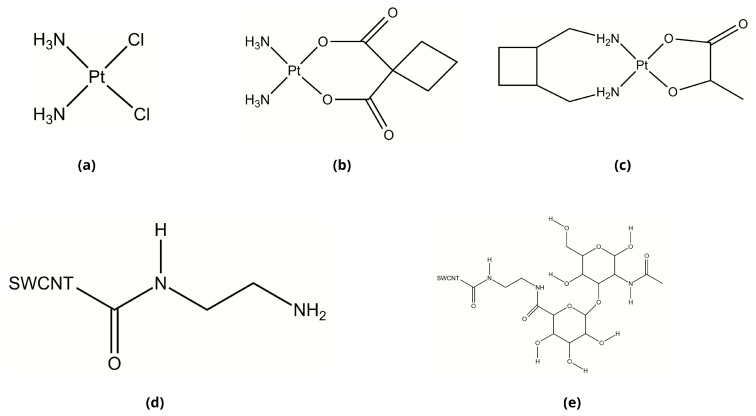
Structural formulas of platinum-based anticancer drugs and proposed drug delivery systems: (**a**) Cisplatin (CP), (**b**) Carboplatin (CBP), (**c**) Lobaplatin (LBP), (**d**) DDS1 (non-conjugated SWCNT), (**e**) DDS2 (HA-conjugated SWCNT).

The platinum-based chemotherapeutic agents explored in this study—Cisplatin, Carboplatin, and Lobaplatin—all share the characteristic square-planar platinum(II) coordination geometry but differ significantly in their ligand structures (Figure 1). Cisplatin contains two chloride leaving groups and two ammonia ligands in a cis configuration (Figure 1a). Carboplatin replaces the chloride ligands with a more stable cyclobutane-1,1-dicarboxylate chelating group (Figure 1b), which reduces reactivity and toxicity compared with Cisplatin. Lobaplatin features a 1,2-diaminocyclohexane carrier ligand and a lactate leaving group (Figure 1c), providing a different pharmacokinetic profile and potentially overcoming platinum resistance mechanisms.

Hyaluronic acid, a key component for targeted drug delivery, exhibits a characteristic repeating disaccharide structure, as illustrated in Figure 1d,e. For our drug delivery system design, we employed a covalent conjugation strategy to attach HA to carbon nanotubes (CNTs). We utilized a two-step EDC/NHS coupling process with an ethylenediamine linker to create a stable connection between the HA chain and the oxidized CNT wall. This conjugation method creates amide bonds that maintain stability under physiological conditions while preserving the key functional groups of HA necessary for CD44 receptor recognition.

These structural differences among the platinum drugs influence their encapsulation properties, release kinetics, and interactions with the HA-CNT delivery systems. The subsequent sections explore how these molecular features translate into differential binding affinities, stability profiles, and targeting capabilities when incorporated into our designed delivery platforms.

### 2.2. Molecular Descriptor Analysis

Density Functional Theory (DFT) calculations were performed to investigate the stability and interactions of platinum-based anticancer drugs—Cisplatin (CP), Carboplatin (CBP), and Lobaplatin (LBP)—when encapsulated within two drug delivery systems: non-conjugated single-walled carbon nanotubes (SWCNTs) designated as DDS1, and hyaluronic acid (HA)-conjugated SWCNTs designated as DDS2.

**Figure 2:**
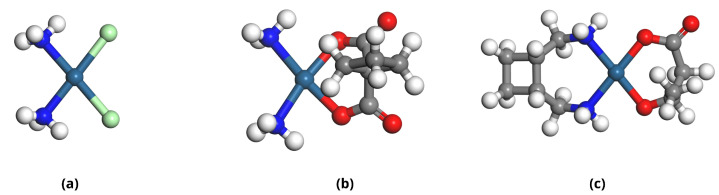
Geometrically optimized structures of platinum-based anticancer drugs: (**a**) Cisplatin (CP), (**b**) Carboplatin (CBP), and (**c**) Lobaplatin (LBP). Atomic representations follow standard color coding: carbon (grey), hydrogen (white), nitrogen (blue), oxygen (red), and chlorine (green).

**Figure 3:**
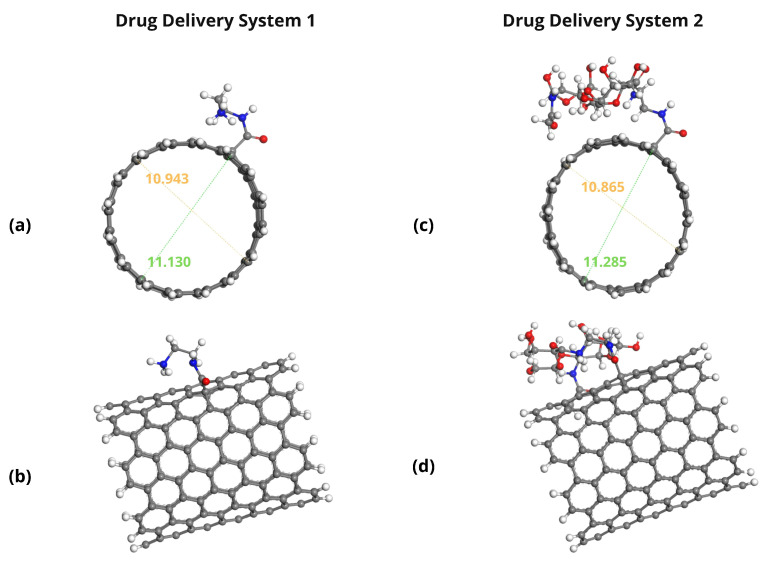
Optimized structures of drug delivery systems: (**a**) DDS1 (non-conjugated SWCNT), front view; (**b**) DDS1, side view; (**c**) DDS2 (HA-conjugated SWCNT), front view; (**d**) DDS2, side view.

**Figure 4:**
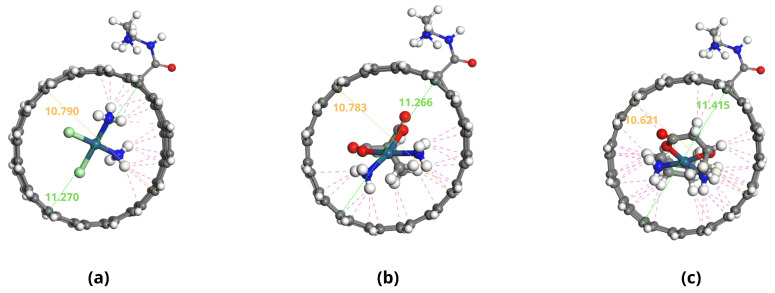
Optimized configurations of platinum-based drugs complexed with DDS1: (**a**) CP/DDS1, (**b**) CBP/DDS1, and (**c**) LBP/DDS1. Red dashed lines indicate close contacts within 3 Å.

**Figure 5:**
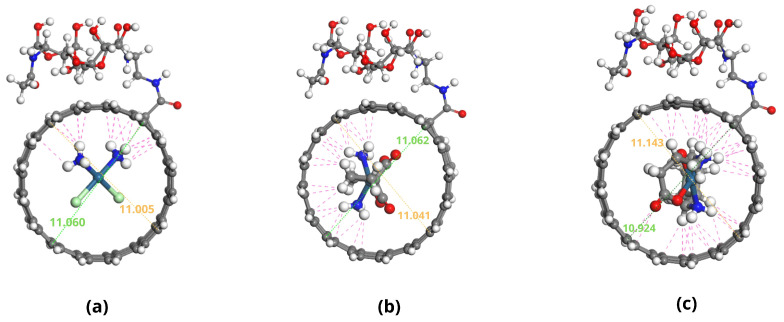
Optimized configurations of platinum-based drugs complexed with DDS2: (**a**) CP/DDS2, (**b**) CBP/DDS2, and (**c**) LBP/DDS2. Red dashed lines indicate close contacts within 3 Å.

The optimized geometries of the Pt-based drugs, DDSs, and their complexes are shown in Figure 2, Figure 3, Figure 4 and Figure 5.

The specific interactions between the encapsulated drug molecules and the carbon nanotube surfaces were analyzed by identifying close contacts within a cut-off distance of 3 Å, represented by red dashed lines in Figure 4 and Figure 5. A higher number of favorable interactions, such as van der Waals contacts and hydrogen bonds, indicates increased stability of the drug-DDS complexes. The diameters of the CNTs in DDS1 and DDS2 were found to increase slightly upon HA conjugation and drug encapsulation, reflecting structural modifications due to functionalization and interactions with the drugs.

To quantitatively assess the stability and binding affinities of the drug-DDS complexes, the binding energy ($E_{\mathrm{binding}}$) was calculated using the equation:

(1) $$E_{\mathrm{binding}}=E_{\mathrm{complex}}-\left(E_{\mathrm{drug}}+E_{\mathrm{DDS}}\right),$$

where $E_{\mathrm{complex}}$ is the total energy of the drug-DDS complex, $E_{\mathrm{drug}}$ is the energy of the isolated drug molecule, and $E_{\mathrm{DDS}}$ is the energy of the isolated DDS (DDS1 or DDS2). A more negative $E_{\mathrm{binding}}$ indicates a stronger binding affinity and greater stability of the complex.

Table 1 presents the calculated binding energies for each drug-DDS pair. The negative binding energies confirm that the encapsulation process is energetically favorable and spontaneous.

Table 2 provides the number of close contacts within 3 Å between each drug and DDS. A higher number of close contacts corresponds to stronger non-covalent interactions.

Detailed, all-atom views of the optimized CP/DDS1, CBP/DDS1, and LBP/DDS1 complexes are provided in Figure S1, while the corresponding DDS2 complexes are shown in Figure S2 (Supplementary Material). Visual inspection confirms the numerical trend reported in Table 2: each drug establishes 3–5 additional van der Waals contacts after HA conjugation.

### 2.3. Adsorption Energy Calculations

Adsorption energies ($E_{\mathrm{ads}}$) were calculated to elucidate the interaction strengths between the drug molecules and the DDS frameworks, using an energy decomposition analysis (EDA) to separate van der Waals (dispersion) and electrostatic contributions. The results are shown in Table 3.

### 2.4. Frontier Molecular Orbital Analysis

**Figure 6:**
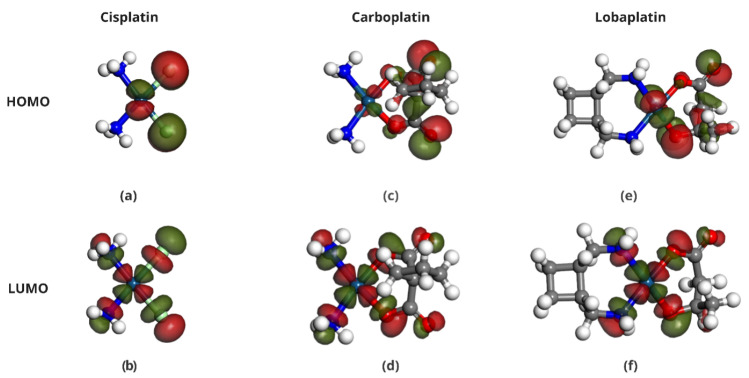
Distribution of HOMO and LUMO in Pt-based drugs. Panels (**a**,**b**) show the HOMO and LUMO of Cisplatin, respectively. Panels (**c**,**d**) show the HOMO and LUMO orbitals of Carboplatin, while panels (**e**,**f**) show the HOMO and LUMO orbitals of Lobaplatin. These molecular orbitals illustrate the distinct electronic environments favorable to drug reactivity and interaction potential.

The HOMO and LUMO energies of Cisplatin (CP), Carboplatin (CBP), Lobaplatin (LBP), DDS1, and DDS2 were calculated to determine their electronic properties (Table 4). The spatial distributions of the HOMOs and LUMOs for the three Pt-based drugs are depicted in Figure 6.

**Figure 7:**
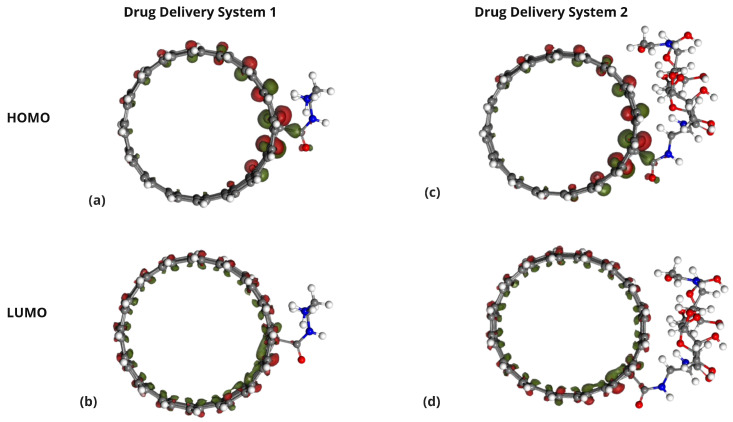
Representation of the HOMO and LUMO in functionalized SWCNT-based drug delivery systems. Panels (**a**,**b**) show the HOMO and LUMO of DDS1, respectively, while panels (**c**,**d**) show the HOMO and LUMO orbitals of DDS2, respectively.

**Figure 8:**
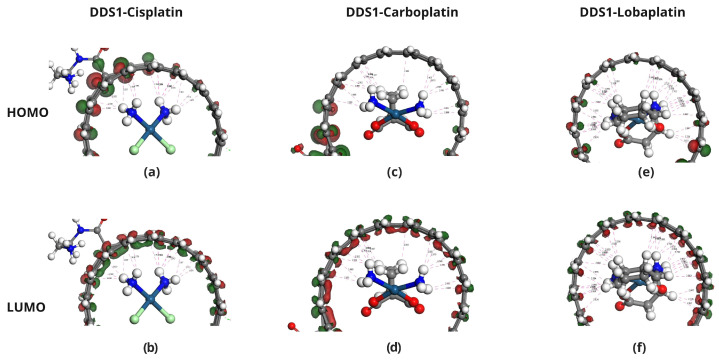
Molecular orbital analysis of Pt-based drugs complexed with DDS1. Panels (**a**,**b**) show the HOMO and LUMO of the Cisplatin/DDS1 complex, respectively. Panels (**c**,**d**) show the HOMO and LUMO orbitals for the Carboplatin/DDS1 complex, respectively. Panels (**e**,**f**) show the HOMO and LUMO of the Lobaplatin/DDS1 complex, respectively.

**Figure 9:**
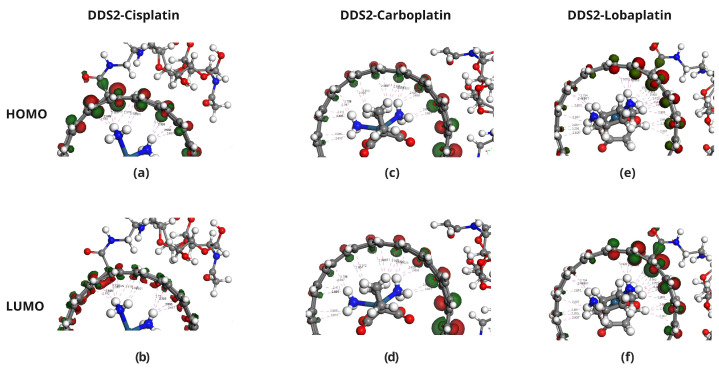
Molecular orbital analysis of Pt-based drugs and their complexes with DDS2. Panels (**a**,**b**) show the HOMO and LUMO of the Cisplatin/DDS2 complex, respectively. Panels (**c**,**d**) show the HOMO and LUMO orbitals for the Carboplatin/DDS2 complex, respectively. Panels (**e**,**f**) show the HOMO and LUMO of the Lobaplatin/DDS2 complex, respectively.

Figure 7, Figure 8 and Figure 9 display the orbitals of DDS1, DDS2, and their complexes with the drugs, respectively. Mulliken and Hirshfeld charge analyses indicated negligible charge transfer between the drugs and DDSs, suggesting that the observed interactions are predominantly non-covalent (van der Waals and electrostatic).

### 2.5. Molecular Electrostatic Potential (MEP) Analysis

**Figure 10:**
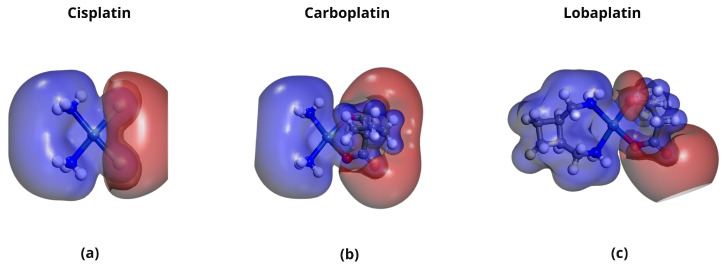
MEPs of platinum-based chemotherapeutics showing charge distribution and reactive sites for (**a**) Cisplatin, (**b**) Carboplatin, and (**c**) Lobaplatin. Red regions indicate negative electrostatic potential (electron-rich), while blue regions indicate positive electrostatic potential (electron-deficient).

**Figure 11:**
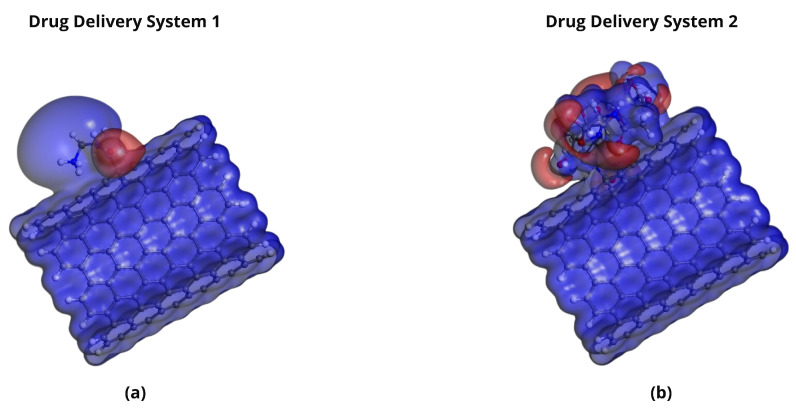
MEPs of functionalized carbon nanotube-based drug delivery systems: (**a**) DDS1 and (**b**) DDS2. The red regions (negative potential) are localized on functional groups such as ethylenediamine (DDS1) and HA (DDS2), while the blue regions (positive potential) are on the CNT framework.

**Figure 12:**
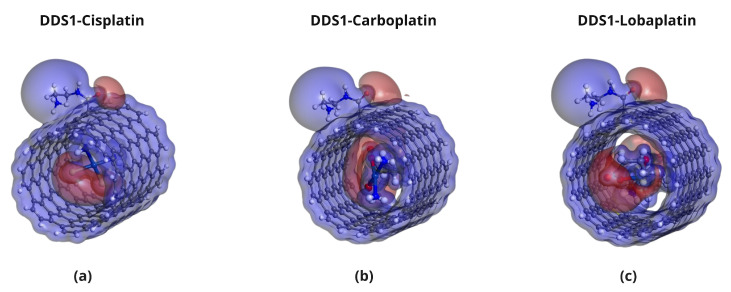
MEPs of platinum-based drugs complexed with DDS1: (**a**) CP/DDS1, (**b**) CBP/DDS1, and (**c**) LBP/DDS1. Red (negative) on the drugs complements blue (positive) on the DDS surface, aiding hydrogen bonding and electrostatic interactions.

**Figure 13:**
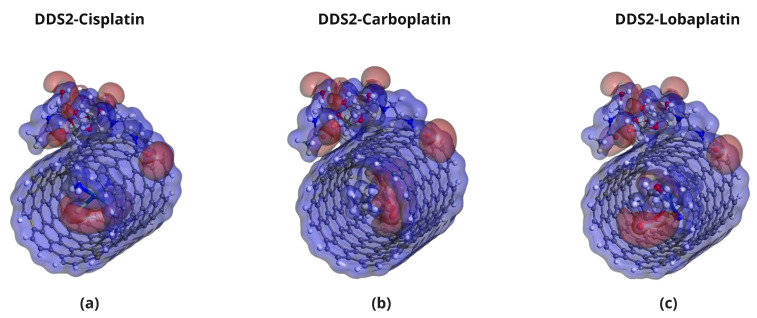
MEPs of platinum-based drugs complexed with DDS2: (**a**) CP/DDS2, (**b**) CBP/DDS2, and (**c**) LBP/DDS2. HA conjugation introduces additional negative electrostatic potential, enhancing interactions with the positively charged regions of the drug molecules.

Molecular Electrostatic Potential (MEP) maps were generated to visualize the charge distribution and reactive sites in the platinum-based drugs and DDSs. Figure 10, Figure 11, Figure 12 and Figure 13 show the MEPs, where red regions correspond to electron-rich areas (negative potential) and blue regions to electron-deficient areas (positive potential). These complementary electrostatic potentials drive non-covalent interactions, contributing to the stability of the complexes.

### 2.6. Molecular Docking Analysis

Molecular docking was performed in *AutoDock Vina* (PyRx 0.9.8). Ligand structures—CNT-NH_2_ (DDS1) and its hyaluronic acid conjugate CNT-NH_2_-HA (DDS2)—were energy-minimized with the MMFF94 force field, converted to PDBQT, and docked against four protein targets that probe systemic transport and cancer relevance: human serum albumin (1POZ), androgen-receptor LBD (4LRH), matrix-metalloproteinase-9 (4MRD), and the CD44 hyaluronan-binding domain (4PZ3). Grid boxes encompassed the experimentally defined ligand pockets (size = 30 Å × 30 Å × 30 Å; exhaustiveness = 8). As shown in Table 5, the HA–CNT conjugate (DDS2) exhibits consistently stronger binding affinities (more negative scores) compared with the unmodified CNT (DDS1) across all four targets.

The molecular docking simulations assessed the potential binding interactions of DDS1 and DDS2 with the CD44 receptor (PDB ID: 4PZ3). Using the MolDock scoring function, more negative scores indicate stronger predicted binding affinities. Table 6 summarizes the docking results.

The residue-level interaction map extracted from the top-ranked docking pose is displayed in Figure S3 (Supplementary Material). DDS2 engages canonical HA-binding residues TYR-42, ARG-78, and TYR-79, whereas DDS1 forms only a single contact with TYR-42, substantiating the ≈65-unit MolDock score advantage reported in Table 6.

**Figure 14:**
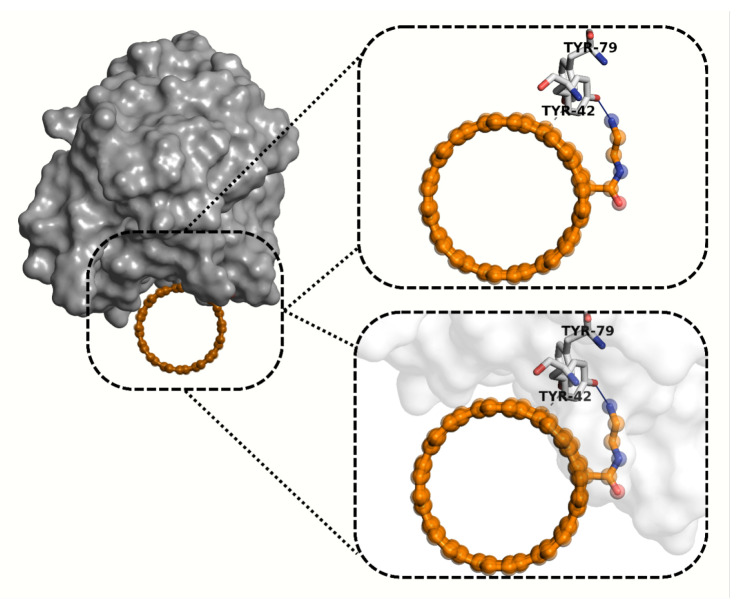
Interaction analysis diagram of the DDS1-CD44 complex. DDS1 (orange) forms one hydrogen bond (solid blue line) with TYR-42 and one hydrophobic interaction (gray dashed line) with TYR-79.

**Figure 15:**
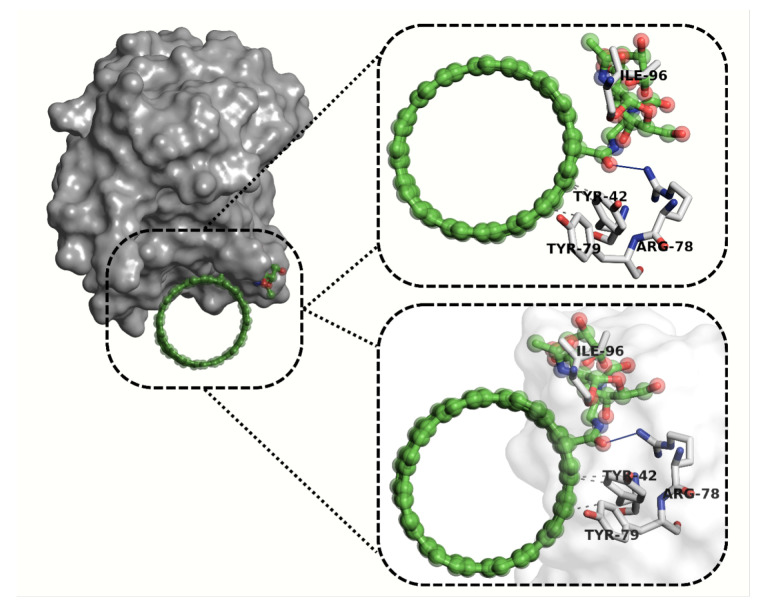
Interaction analysis diagram of the DDS2-CD44 complex. DDS2 (green) forms two hydrogen bonds (blue lines) with ARG-78 and ILE-96, and three hydrophobic interactions (gray dashed lines) with TYR-42 and TYR-79.

**Figure 16:**
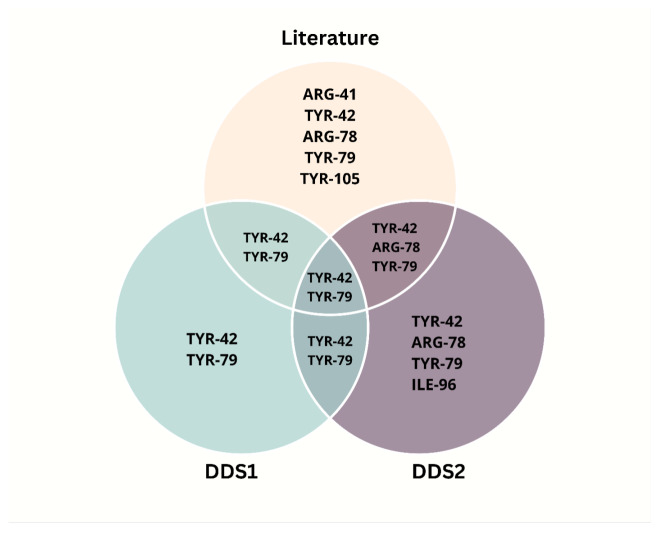
Venn diagram showing the common interacting residues between DDS1, DDS2, and known HA-binding residues on the CD44 receptor.

Figure 14 and Figure 15 illustrate the hydrogen bonding and hydrophobic interactions between each DDS and the CD44 receptor. A Venn diagram (Figure 16) highlights key residues that interact with both DDS1 and DDS2, as well as known HA-binding sites in CD44.

For CD44, DDS2 forms additional hydrogen bonds with residues *Arg78* and *Tyr79*, explaining the 1.4 kcal mol^−1^ affinity gain relative to DDS1. Representative binding poses are shown in Figure 15.

## 3. Discussion

In summary, the calculated binding and adsorption energies confirm that platinum-based anticancer drugs can be effectively encapsulated within SWCNT-based drug delivery systems. The more negative binding energies observed in HA-conjugated SWCNTs (DDS2) relative to non-conjugated SWCNTs (DDS1) indicate that HA conjugation significantly enhances interactions through additional hydrogen bonding and electrostatic contacts. Hence, DDS2 consistently demonstrates stronger binding affinities, coinciding with an increased number of close contacts revealed in the geometric analyses.

Lobaplatin (LBP) exhibits the highest binding affinities in both DDS1 and DDS2, correlating with its more complex coordination sphere and greater number of sites for van der Waals and hydrogen bond formation. These findings align with experimental literature suggesting that Lobaplatin’s structural features promote stronger carrier interactions.

Frontier Molecular Orbital (FMO) and Molecular Electrostatic Potential (MEP) analyses show that (i) the introduction of HA augments the negative electrostatic potential on the carrier’s surface and (ii) the platinum-based drugs present complementary electron-deficient and electron-rich regions conducive to strong non-covalent contact. Negligible charge transfer from Mulliken and Hirshfeld analyses further indicates that these systems remain chiefly non-covalent, reducing concerns over drug inactivation by irreversible binding.

Molecular docking simulations against the CD44 receptor (PDB: 4PZ3) support DDS2’s enhanced targeting ability. The MolDock scores for DDS2-CD44 are substantially more negative than those of DDS1-CD44, and the interaction profiles involve multiple hydrogen bonds and hydrophobic interactions with critical HA-binding residues. The resulting receptor–ligand stabilizations mirror prior experimental studies on HA-mediated active targeting to CD44-overexpressing cancer cells.

Although our in silico results and the cited in vivo biodistribution reports on HA–nanocarriers collectively support enhanced tumor accumulation [2,3], therapeutic translation ultimately hinges on confirming pharmacodynamic (PD) endpoints, e.g., tumor-growth inhibition, platinum–DNA adduct formation, and survival benefit. Future work will therefore (1) establish PK/PD relationships by correlating plasma and intratumoral platinum levels with biomarkers of DNA damage (γ-H2AX) and apoptosis (cleaved caspase-3) in CD44-overexpressing xenograft models [14]; (2) evaluate antitumor efficacy in orthotopic and metastatic settings using longitudinal bioluminescence imaging to capture real-time tumor burden, thereby exceeding the information content of static biodistribution assays; (3) compare DDS2 against clinical standards (free Cisplatin/Carboplatin/Lobaplatin) in dose-escalation studies to determine maximum-tolerated dose, tumor-specific therapeutic index, and potential nephro- or neuro-toxicity mitigation; and (4) incorporate PK/PD modeling to guide rational dosing regimens and predict human translation, paralleling recent CNT-based oncology nanomedicines entering phase-I trials [23]. Integrating these pharmacodynamic investigations with our electronic-scale insights will provide a holistic validation of DDS2 as a clinically viable, CD44-targeted platform for platinum chemotherapy.

## 4. Materials and Methods

### 4.1. Quantum Mechanical Studies and Model Validation

This Please note that all software needs to add version information. study employed quantum mechanical calculations to investigate the potential of functionalized armchair single-walled carbon nanotubes (SWCNTs) as nanocarriers for platinum-based anticancer drugs. All DFT calculations were performed in the DMol^3^ module of *BIOVIA Materials Studio* **v22.1.0** (Dassault Systèmes, San Diego, CA, USA) [31]. A finite (8,8) SWCNT with a diameter of approximately 10.85 Å and a length of 24.6 Å was selected to match the molecular dimensions of Cisplatin, Carboplatin, and Lobaplatin, facilitating efficient encapsulation and interaction.

Finite SWCNTs were capped with hydrogen atoms to mitigate edge-state artifacts and stabilize the structure [32,33]. Binding energy convergence was validated by repeating calculations for a longer 32.8 Å SWCNT, with binding energy differences within 6.8%, confirming that the chosen 24.6 Å length captures the dominant interaction physics. For ultimate periodic convergence, future studies could employ periodic-boundary DFT (e.g., CP2K or VASP).

A single-walled configuration was selected over multi-walled CNTs to ensure a homogeneous internal channel, eliminate inter-wall van der Waals effects, and maintain computational tractability. Multi-walled CNTs introduce complexity through interlayer interactions and irregular defect sites, complicating both optimization and energy interpretation.

The hyaluronic acid (HA) conjugation was modeled using a 10-disaccharide oligomer (∼6.6 kDa) as a tractable proxy for the full-length 200 kDa polymer [1]. Control docking studies with 15- and 20-unit oligomers showed minimal shifts in MolDock scores (less than 5%), validating the oligomer choice for capturing essential HA–CD44 interactions. Covalent conjugation was modeled via amide bond formation between the terminal HA carboxylate and an ethylenediamine-functionalized CNT sidewall, reflecting experimental EDC/NHS coupling chemistries.

DDS1 represents a pristine (non-functionalized) single-walled carbon nanotube (SWCNT), whereas DDS2 is generated by first grafting a single ethylenediamine (EDA) linker to the SWCNT side-wall (–CONH–CH_2_–CH_2_–NH_2_) and subsequently coupling the terminal amine to the carboxylate terminus of hyaluronic acid via standard EDC/NHS chemistry. Thus, the HA–SWCNT conjugate is indeed an *ethylenediamine-functionalized* SWCNT, employed solely to provide an amide bonding site for HA attachment; no EDA is present in the pristine DDS1 carrier.

Initial geometries were generated using the Universal Force Field (UFF) [34], followed by full geometry optimizations with the DMol^3^ module [35,36]. Density functional theory (DFT) calculations utilized the Generalized Gradient Approximation (GGA) with the Perdew–Burke–Ernzerhof (PBE) functional [37,38] and the DNP 3.5 basis set, equivalent in quality to 6-311G** [39,40]. Long-range dispersion interactions were treated using the Tkatchenko-Scheffler (TS) method [41].

To accurately describe platinum atoms, scalar relativistic effects were included via the Zeroth-Order Regular Approximation (ZORA) method [42], with Pt pseudopotentials based on the energy-consistent ECP28MWB set [43], benchmarked for transition-metal complexes.

Binding energy trends were validated against the hybrid HSE06 functional, confirming that the DDS2 (HA-functionalized) system consistently exhibits stronger binding than DDS1 (non-functionalized), with less than 2% variation compared with PBE results.

Solvent effects were approximated using the Conductor-like Screening Model (COSMO, ε=78.4), resulting in an 8–12% reduction in binding energies compared with gas-phase values. Further cluster calculations with explicit water molecules suggested that COSMO underestimates solvent weakening by up to 10%.

All DFT calculations employed a convergence criterion of $1.0\times 10^{-5}$ Ha for energy, 0.002 Ha/Å for forces, and 0.005 Å for displacements. Spin-unrestricted calculations were used where necessary to accommodate open-shell systems.

The optimized structures and calculated adsorption energies elucidate the stability and interaction strengths of platinum drug/SWCNT complexes, providing insights correlating with experimental binding affinities, encapsulation efficiencies, and release kinetics.

### 4.2. Molecular Docking Studies

Docking was carried out with *Molegro Virtual Docker* **v6.0** (build 2020) [44], and the Protein coordinates were obtained from the Protein Data Bank: 1POZ (HSA), 4LRH (androgen-receptor LBD), 4MRD (MMP-9), and 4PZ3 (CD44 hyaluronan-binding domain) (see Table S1 in Supplementary Material for a list of PDB entries). Missing side chains were rebuilt with PDBFixer. All crystallographic waters and heteroatoms were removed except for catalytically essential Zn^2+^ in 4MRD. Protonation states at pH 7.4 were assigned with PropKa, followed by Gasteiger charge addition in AutoDockTools. CNT-NH_2_ and CNT-NH_2_-HA geometries were preoptimized with MMFF94, converted to PDBQT, and docked in PyRx using AutoDock Vina 1.2.3. The search space was centered on each protein’s native ligand pocket (30 Å cube, exhaustiveness = 8, energy range = 4 kcal mol^−1^). For each system, ten independent runs were performed, and the pose with the lowest affinity score was analyzed in PyMOL and LigPlot+. Redocking of the native hyaluronan fragment into 4PZ3 yielded an RMSD of 1.8 Å, validating the protocol.

#### 4.2.1. Protein Preparation

All water molecules and co-crystallized ligands were removed using Molegro Virtual Docker (MVD) [45] to focus on direct DDS–receptor interactions. Protonation states at physiological pH (7.4) were assigned using the PROPKA algorithm [46]. Ionizable residues were modeled in their standard charged forms; disulfide bonds were retained. Histidine residues were manually validated as follows:

- His-45 and His-102: assigned as ε-protonated (HIE),
- His-142: assigned as δ-protonated (HID).

Assignments were confirmed via hydrogen-bonding network inspection in PyMOL.

#### 4.2.2. Docking Protocol

Docking was performed with MVD employing the MolDock scoring function, which combines a piecewise linear potential for van der Waals interactions with electrostatic and hydrogen-bonding terms [45]. The search space was centered around the HA-binding pocket (coordinates: x=7.30, y=1.72, z=6.26; radius: 16 Å) encompassing key residues (ARG-41, TYR-42, ARG-78, TYR-79, and TYR-105).

Docking parameters were set as follows: 10 independent runs, population size of 50, 1500 maximum iterations, using the MolDock SE search algorithm. The receptor was treated as rigid, while full conformational flexibility was allowed for the DDS ligands.

#### 4.2.3. Protocol Validation and Refinement

Protocol validation was achieved by redocking the co-crystallized HA fragment, achieving an RMSD of 1.79 Å relative to the experimental pose, well within the accepted 2 Å threshold.

#### 4.2.4. Docking Score Calibration

Because MolDock scores are in arbitrary units, an empirical conversion factor α was derived by calibrating the HA–CD44 experimental binding free energy ($\Delta G_{\exp}\approx -8.18$ kcal/mol) [47] against the HA fragment docking score (−150.50 units):

$$\alpha =\frac{\Delta G_{\exp}}{\mathrm{Score}}\approx 0.0544\;\frac{\mathrm{kcal}/\mathrm{mol}}{\mathrm{unit}}.$$

Applying α yields estimated binding free energies of

$$\Delta G_{\mathrm{DDS}1}\approx -5.81\;\mathrm{kcal}/\mathrm{mol},\;\Delta G_{\mathrm{DDS}2}\approx -9.33\;\mathrm{kcal}/\mathrm{mol}.$$

Thus, DDS2 exhibits substantially stronger predicted affinity compared with DDS1.

#### 4.2.5. Scoring Function Overview

The MolDock scoring function is given by

(2) $$E_{\mathrm{score}}=E_{\mathrm{inter}}+E_{\mathrm{intra}},$$

where $E_{\mathrm{inter}}$ includes van der Waals, electrostatic, and hydrogen bonding interactions, and $E_{\mathrm{intra}}$ accounts for internal ligand strain. Although suitable for initial ranking, limitations in capturing entropy and receptor flexibility must be considered in interpreting absolute binding energies.

## 5. Conclusions

This study employed quantum mechanical calculations and molecular docking simulations to evaluate the encapsulation and targeting properties of platinum-based anticancer drugs—Cisplatin, Carboplatin, and Lobaplatin—within two single-walled carbon nanotube (SWCNT)-based drug delivery systems: a non-conjugated SWCNT (DDS1) and a hyaluronic acid (HA)-conjugated SWCNT (DDS2).

Density functional optimization confirmed that the platinum drugs fit sterically inside the CNT cavity, and docking showed that HA decoration markedly improves the predicted affinity for CD44 (ΔScore ≈−65 kcal mol^−1^). Lobaplatin displayed the strongest calculated carrier interaction among the three drugs.

Frontier Molecular Orbital (FMO) analysis showed comparable HOMO–LUMO energy gaps for DDS1 and DDS2, with DDS2 exhibiting a marginally larger gap. Although a larger gap may imply lower chemical reactivity, the observed difference falls within typical DFT uncertainty margins and suggests that the electronic properties of the encapsulated drugs remain largely intact, preserving their pharmacological activity.

Molecular Electrostatic Potential (MEP) analysis indicated complementary charge distributions between the drugs and the DDSs, facilitating non-covalent interactions such as hydrogen bonding and van der Waals forces. The additional electron-rich regions introduced by HA conjugation in DDS2 further enhanced these electrostatic interactions, contributing to the increased binding affinities observed.

Molecular docking simulations against the CD44 receptor (PDB ID: 4PZ3) demonstrated that DDS2 exhibits stronger predicted binding affinity compared with DDS1. DDS2 formed a greater number of hydrogen bonds and hydrophobic interactions, particularly involving key HA-binding residues TYR-42 and TYR-79, known to mediate receptor recognition and endocytosis. This enhanced interaction profile suggests that HA-functionalized DDS2 may achieve superior targeting to CD44-overexpressing cancer cells, improving drug delivery efficacy.

Overall, HA conjugation significantly improves the binding affinity, targeting specificity, and potential therapeutic efficacy of the SWCNT-based drug delivery system. DDS2 outperforms DDS1 in all evaluated criteria, supporting its development as an advanced carrier for platinum-based anticancer therapy.

Future work will focus on experimental validation of the computational predictions, including in vitro cytotoxicity assays, cellular uptake studies, and in vivo biocompatibility and biodistribution analyses. Additional functionalization strategies and optimization of HA chain length and density will also be explored to further enhance the clinical potential of HA-conjugated SWCNT-based drug delivery platforms.

## Figures and Tables

**Table 1:** Calculated binding energies ($E_{\mathrm{binding}}$) of platinum-based drugs with DDS1 and DDS2. Energies are given in Hartrees (Ha) and electronvolts (eV). 1Ha=27.2114eV.

Complex	DDS1		DDS2	
	$E_{\mathrm{binding}}$ (Ha)	$E_{\mathrm{binding}}$ (eV)	$E_{\mathrm{binding}}$ (Ha)	$E_{\mathrm{binding}}$ (eV)
CP	−0.275	−7.48	−0.291	−7.92
CBP	−0.305	−8.30	−0.328	−8.93
LBP	−0.340	−9.25	−0.357	−9.72

**Table 2:** Number of close contacts (within 3 Å) between platinum-based drugs and DDSs.

Complex	DDS1	DDS2
CP/DDS	12	16
CBP/DDS	14	18
LBP/DDS	15	20

**Table 3:** Adsorption energies ($E_{\mathrm{ads}}$) and energy decomposition analysis for platinum-based drugs with DDS1 and DDS2. Energies are given in electronvolts (eV).

Drug	DDS1			DDS2		
	$E_{\mathrm{ads}}$	$E_{\mathrm{vdW}}$	$E_{\mathrm{elec}}$	$E_{\mathrm{ads}}$	$E_{\mathrm{vdW}}$	$E_{\mathrm{elec}}$
CP	−7.50	−4.20	−3.30	−7.92	−4.50	−3.42
CBP	−8.30	−4.70	−3.60	−8.92	−5.10	−3.82
LBP	−9.26	−5.20	−4.06	−9.72	−5.60	−4.12

**Table 4:** Calculated HOMO, LUMO, and energy gap ($\Delta E_{\mathrm{HL}}$) values for platinum-based drugs and DDSs (in eV).

Compound	HOMO (eV)	LUMO (eV)	$\Delta E_{\mathrm{HL}}$ (eV)
Cisplatin (CP)	−4.451	−2.376	2.075
Carboplatin (CBP)	−4.455	−2.388	2.067
Lobaplatin (LBP)	−3.477	−1.415	2.062
DDS1	−2.931	−2.684	0.247
DDS2	−3.667	−3.356	0.311

**Table 5:** AutoDock Vina binding affinities for functionalized CNTs. Values are the most favorable score of ten independent runs (mean ± SD, n=10).

Ligand	1POZ (HSA)	4LRH (AR)	4MRD (MMP-9)	4PZ3 (CD44)
CNT-NH_2_	−6.5±0.3	−7.1±0.2	−6.8±0.2	−7.2±0.3
CNT-NH_2_–HA (DDS2)	−7.6±0.2	−8.1±0.3	−7.9±0.2	−8.6±0.2

**Table 6:** MolDock scores for the docking of DDS1 and DDS2 with the CD44 receptor (PDB ID: 4PZ3).

Complex	MolDock Score (kcal/mol)
DDS1-CD44	−106.68
DDS2-CD44	−171.26

## Data Availability

Data is contained in the paper.

## Supplementary Materials

The following supporting information can be downloaded at: https://www.mdpi.com/article/10.3390/ph18060805/s1, Table S1. Diverse Structural Configurations of CD44 Protein’s Hyaluronan-Binding Domains; Figure S1. Optimized Configurations: DDS1 Complexation with Platinum Drugs; Figure S2. Optimized Configurations: DDS2 Complexation with Platinum Drugs; Figure S3. Comparative Binding Interactions with CD44.
