# Supplementary material for: DFT and Molecular Docking Study of HA-Conjugated SWCNTs for CD44-Targeted Delivery of Platinum-Based Chemotherapeutics
Source: Pharmaceuticals (Basel). 2025 May 27;18(6):805. doi: 10.3390/ph18060805 (PMC12195731; doi:10.3390/ph18060805)
Supplement: Supplementary file 1 [file pharmaceuticals-18-00805-s001.zip › pharmaceuticals-3635389-supplementary.pdf]

Supplementary Material for: DFT and Molecular Docking Study of HA-Conjugated SWCNTs for CD44-Targeted Delivery of Platinum-Based Chemotherapeutics

Muhammad Uzair Khan, Ishrat Jabeen, Abdulhamid Althagafi,
Muhammad Umar Farooq, Moussab Harb, Bassim Arkook

Table S1. Diverse Structural Configurations of CD44 Protein's Hyaluronan-Binding Domains

Table S1. Diverse Structural Configurations of CD44 Protein's Hyaluronan-Binding Domains, as Revealed by Protein Data Bank Entries.

PDB-ID	Organism	Publication Date	Resolution	Conformation
1POZ	Homo sapiens	2004-03-16	SOLUTION NMR	Hyaluronan binding domain
1UUH	Homo sapiens	2004-03-04	X-RAY (2.20 Å)	Hyaluronan binding domain
2I83	Homo sapiens	2006-11-21	SOLUTION NMR	Hyaluronan-binding domain (ligand-bound)
4PZ3	Homo sapiens	2014-09-17	X-RAY (1.08 Å)	CD44 hyaluronan domain + peptides
4PZ4	Homo sapiens	2014-09-17	X-RAY (1.60 Å)	CD44 hyaluronan domain (new space group)

Figure S1. Optimized Configurations: DDS1 Complexation with Platinum Drugs

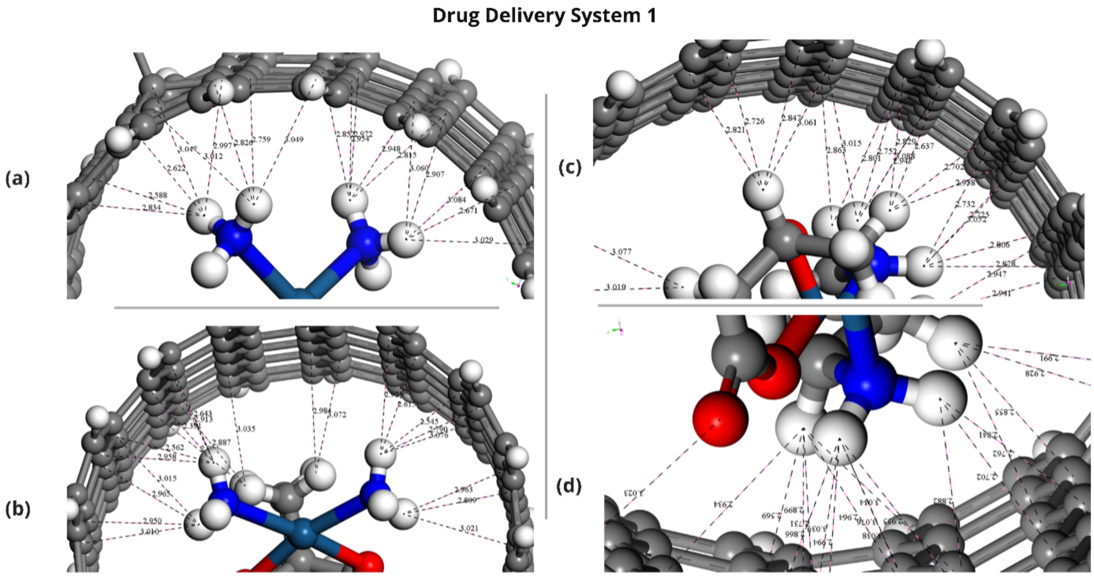

Figure S1. Computationally optimized molecular configurations of three platinum-based drugs complexed with DDS1. (a) Cisplatin, (b) Carboplatin, and (c,d) Lobaplatin (two views). Visualized with BIOVIA Material Studio.

Figure S2. Optimized Configurations: DDS2 Complexation with Platinum Drugs

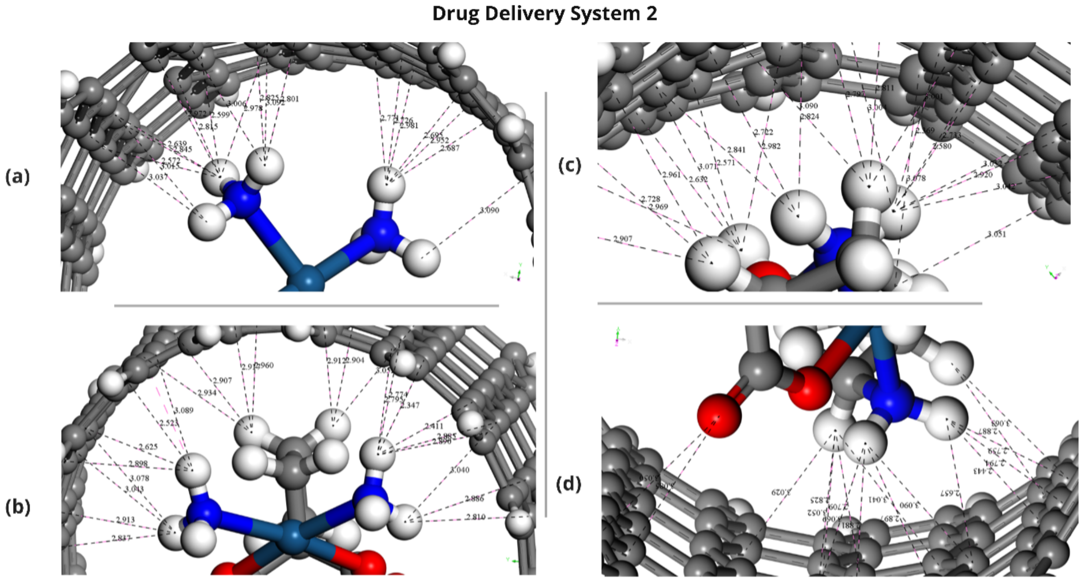

Figure S2. Optimized molecular configurations of three platinum-based drugs complexed with DDS2. (a) Cisplatin, (b) Carboplatin, and (c,d) Lobaplatin (two views).

Figure S3. Comparative Binding Interactions with CD44

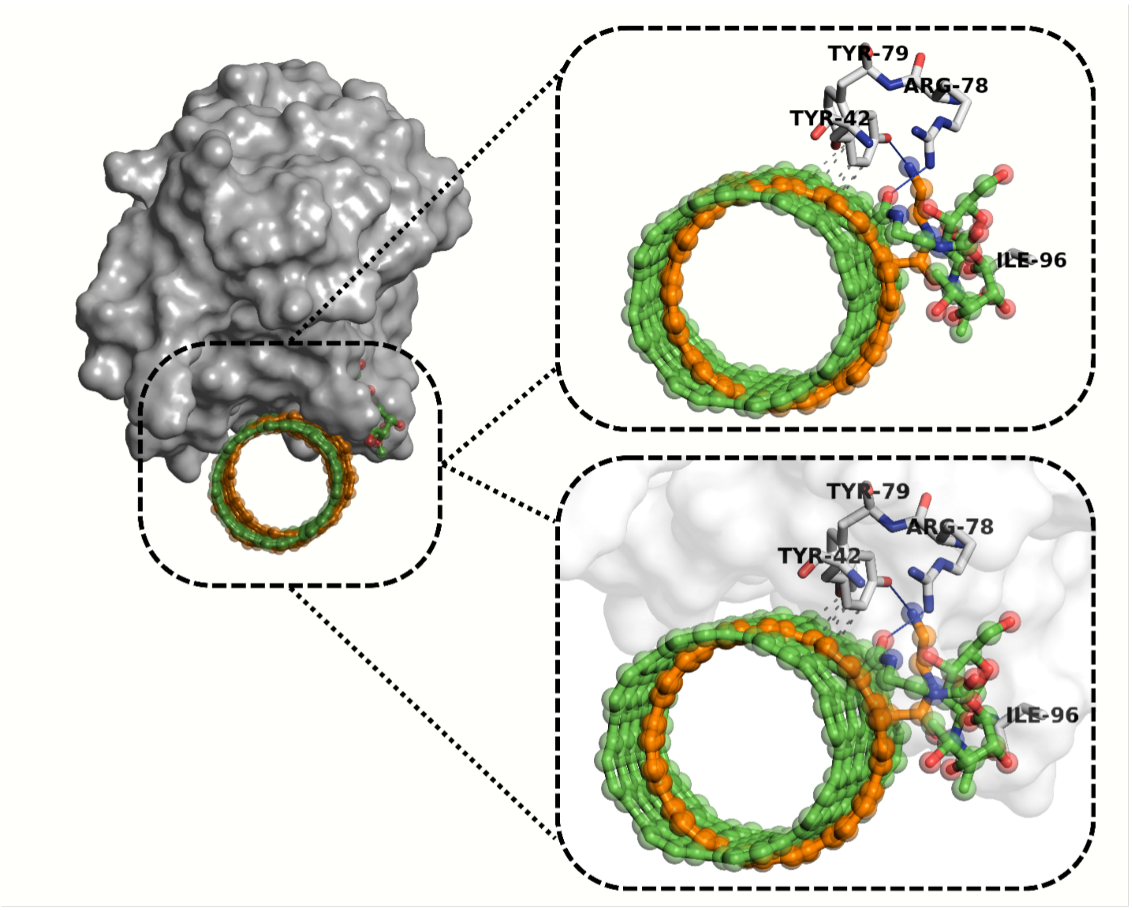

Figure S3. Comparative analysis of DDS1 (orange) and DDS2 (green) binding interactions with the amino acid residues of CD44 receptor (PDB ID: 4PZ3, Chain A).
